# Obituary

**Published:** 1873-03

**Authors:** 


OBITUARY.—We have to record the death on February 23d, of Dr. URIAH G. BIGELOW, of Albany, N. Y. Dr. Bigelow was one of the prominent physicians of Albany, and was loved and respected by all. At the time of his death he was fifty-one years of age, and had been an invalid for some time. He was a permanent member of the New York State Medical Society and was connected with the Albany Medical College. Dr. Bigelow occupied a high position in the profession and his death will be a loss to the Medical profession of Albany and of the State.

